# A Bioinspired Twin Inverted Multiscale Matched Filtering Method for Detecting an Underwater Moving Target in a Reverberant Environment

**DOI:** 10.3390/s19235305

**Published:** 2019-12-02

**Authors:** Xueli Sheng, Chaoping Dong, Longxiang Guo, Li Li

**Affiliations:** 1 Acoustic Science and Technology Laboratory, Harbin Engineering University, Harbin 150001, China; shengxueli@hrbeu.edu.cn (X.S.); dongchaoping@hrbeu.edu.cn (C.D.); li.li@hrbeu.edu.cn (L.L.); 2 Key Laboratory of Marine Information Acquisition and Security, Ministry of Industry and Information Technology, Harbin Engineering University, Harbin 150001, China; 3 College of Underwater Acoustic Engineering, Harbin Engineering University, Harbin 150001, China

**Keywords:** biosonar, sonar waveform design, underwater target detection, reverberation

## Abstract

To this day, biological sonar systems still have great performance advantages over artificial sonar systems, especially for detection in environments with clutter, strong reverberation, and a low signal to noise ratio (SNR). Therefore, mammal sonar systems, for instance, bats and toothed whales, have many characteristics worth learning from. This paper proposes a bioinspired twin inverted multiscale matched filtering method to detect underwater moving targets. This method can be mainly divided into three parts. Firstly, a hyperbolic frequency modulation (HFM) continuous wave (CW) multiharmonic detection signal was adopted after analyzing signals from bats and dolphins. This signal combines the advantages of CW and HFM signals and has excellent time measurement and speed measurement performance when detecting a moving target. Secondly, the twin inverted waveform was introduced to suppress strong linear reverberation and highlight moving targets. The pulse interval was determined by assessing the reverberation reduction time. Thirdly, when processing echoes, a multiscale matched filtering method was proposed to make use of multiharmonic information and improve detection performance. Finally, a channel pool experiment was carried out to test the performance of the proposed method. The experimental result demonstrates that the proposed method has better performance when detecting a moving target in a reverberant environment compared to the conventional matched filtering method. Related results can be applied to small underwater platforms or sensor network platforms for target detection and coastal defense applications.

## 1. Introduction

Applications of artificial sonar systems, for instance, underwater communication, detection, and navigation, are increasingly facing complex environments [1]. However, mammals in the natural ecology, such as cetaceans, bats, etc., can make good use of acoustic detection methods to achieve positioning, obstacle avoidance, detection, and communication functions. These mammals have a high degree of similarity in the relevant 200 groups of genes [2,3,4]. The environmental adaptation allows mammals to learn and evolve solutions for complex underwater environments [5].

Biosonar is a promising field of technology, focusing on the physiological and behavioral analysis of sonar utilization during the predation and socialization of bats and toothed whales, summarizing its advantages in communication, detection, navigation, and signals processing, and, most importantly, applies waveforms, detection logic, signal processing, and target recognition methods to artificial sonar systems. Experts from various fields have long explored the underwater applications of biosonar. This paper mainly focuses on biosonar target detection. 

As mammal sounds are ubiquitous in nature, they can be used as carriers for covert detection signals. Jiang designed a target range and velocity measurement combination (RVMC) method by using two true sperm whale call pulses which had an excellent range resolution (RR) and large Doppler tolerance [6]. They used true call pulses to ensure the camouflage ability of sonar waveforms. Wang simulated the feasibility of a whale-inspired signal for covert active sonar detection based on humpback whale songs. The simulation results demonstrated that the whale-inspired signal had a good range and velocity resolution when dealing with reverberation [7]. 

Biosonar waveforms may contain some clues which can improve biosonar performance, for instance, the time-frequency distribution, energy distribution, and beam pattern. Chris Capus et al. found that the echolocation signal of dolphins is a broadband, high-frequency narrow-pulse signal with different degrees of overlap in both the time and frequency domains and has a very high time resolution [8]. They used the wideband sonar signal of the wide-nose dolphins to observe the ocean and seabed geomorphology, and combined the click signal of the dolphin with the side-scan sonar to detect buried cable [9]. Paihas used dolphin echoes to study the reconstruction of underwater targets from the perspective of dolphins, and explore underwater detection and imaging from the perspective of dolphins. Related application studies have been used to implement bio-SLAM for the detection, imaging, and identification of buried underwater targets [10]. Based on the analysis of the beam pattern of dolphins, Houser et al. designed a bionic sensing array [11]. The bionic cross-correlation beamforming and adaptive beamforming could achieve a resolution of 5 cm for two targets in shallow water. 

Biosonars are also well-suited to harsh environments, and several features have been identified that may be used to suppress clutter. Inspired by dolphins and whales using a bubble net for hunting [12,13], a twin inverted pulse sonar (TWIPS) was first proposed by Leighton [14], it was utilized to detect and classify targets against clutter by distinguishing between linear and nonlinear scatterers. The experiment result here was promising, and the method can be used to detect linear targets in bubbly water. Later, it was applied to detect targets in ship wakes [15]. The results showed that the method requires high fidelity of the hardware systems in this circumstance. TWIPS provides inspiration for the approach of this article.

The purpose of this paper is to propose a bioinspired and effective method for underwater target detection in reverberant environments. The structure of this paper is as follows: In Section 2, we analyze the bats’ and dolphins’ signals and adopted a bioinspired hyperbolic frequency modulation (HFM)-continuous wave (CW) multiharmonic waveform. In Section 3, a twin inverted multiscale matched filtering method is proposed, where the twin inverted waveform is designed to suppress reverberation and highlight the target, and the multiscale matched filtering is designed to utilize the harmonic information of detection signal and improve the detection performance. Pool test results are provided in Section 4, where the pulse interval determination method is also presented. Section 5 presents the conclusions of the present work.

## 2. Bioinspired Waveform Design

### 2.1. Mammal Sonar Signal Analysis

#### 2.1.1. Waveform

**Figure 1:**
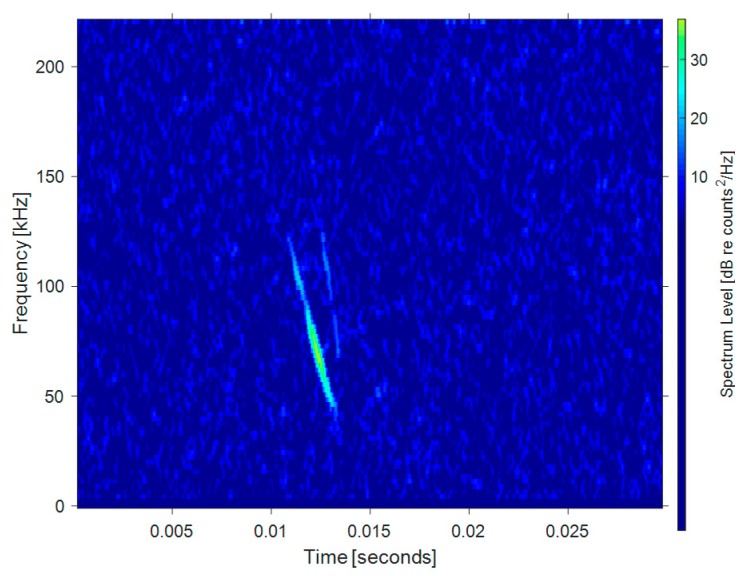
Time-frequency distribution of a wideband FM bat signal.

The waveforms of bat echolocation signals are diverse and species-specific. Different echolocation types are often associated with their predation environment, and some species can even adjust their acoustic signal structures according to changes in environmental conditions [5]. Specifically, the echolocation signals of bats can be mainly divided into three different types [16]:

Wideband FM wave: This type of signal is a very short (only a few millisecond) frequency-modulated (FM) wave, which is the most common wave among bats using echolocation. Such ultrasonic waves are usually short in wavelength, wide in spectrum, have good time resolution, and can be used to quickly determine the direction, distance, and characteristics of the target. Bats can utilize accurate time measurement and binaural time difference for localization [17]. Figure 1 shows the echolocation signal of Bechstein’s bat (*Myotis bechsteinii*). The data below are from Bat Ecology and Bioacoustics Lab, University of Bristol [18]. The signal is a steep modulated down-chirp FM wave, with a main frequency ranging from 40–100 kHz and duration time of about 2 ms.

**Figure 2:**
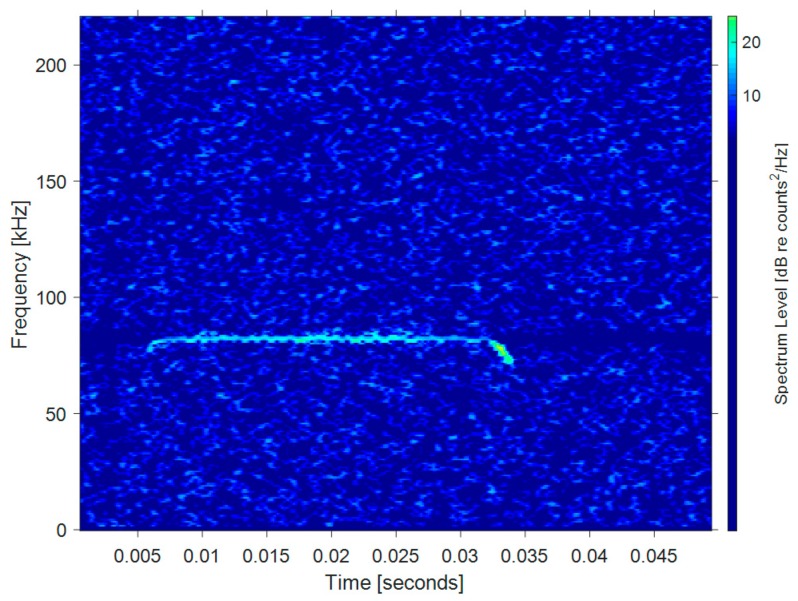
Time-frequency distribution of a long constant frequency-frequency modulated (CF-FM) bat signal.

Long constant frequency (CF) and FM wave: This ultrasonic signal is mainly composed of a constant frequency (CF or CW) component and a tail of an FM component. Bats that make use of this wave use the CF component to measure the Doppler shift of the target to determine the relative velocity. At the same time, the Doppler shift is also used to compensate for the processing of the FM signal for a more accurate analysis of the echo. CF-FM bats usually hunt prey in complex environments where it is extremely difficult to detect prey with a simple FM signal. Such bats gradually reduce the CF component in their pulses and increase the repetition rate while chasing prey. In some species, the last pulse consists almost entirely of FM components. Figure 2 shows the echolocation signal of a greater horseshoe bat (*Rhinolophus ferrumequinum*). The signal is comprised of a long CF component in the middle and short FM on the two sides. The duration is about 30 ms and the frequency bandwidth is relatively lower than what is found for Bechstein’s bats.

**Figure 3:**
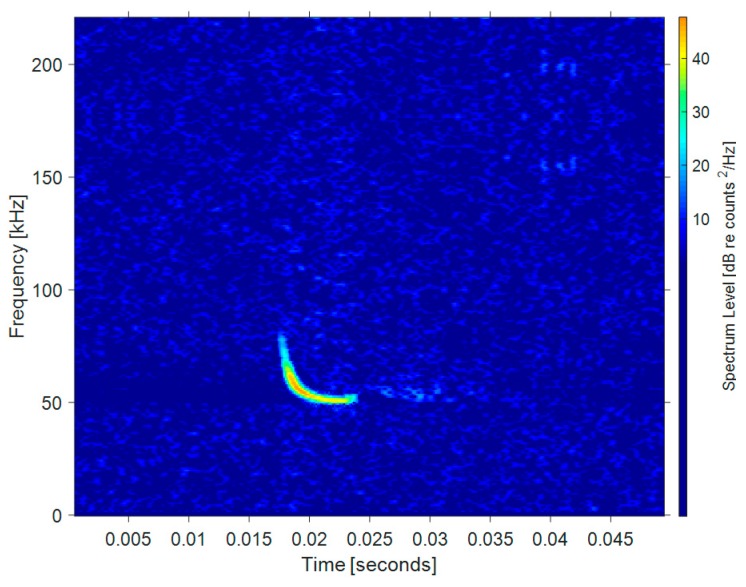
Time-frequency distribution of a short CF-FM bat signal.

Short CF and FM wave: This kind of bat signal contains a short CF component and a FM component, which is considered to be a primitive evolutionary type. Short CF-FM bats may use Doppler information to some extent. Unlike long CF-FM bats, short CF-FM bats may use CF components to detect targets, however, when chasing prey, most of the information about the target is obtained from FM scans. Figure 3 shows the echolocation signal of *Pipistrellus pygmaeus*, where the signal starts with a short FM signal and ends with a short CF component.

#### 2.1.2. Harmonics

**Figure 4:**
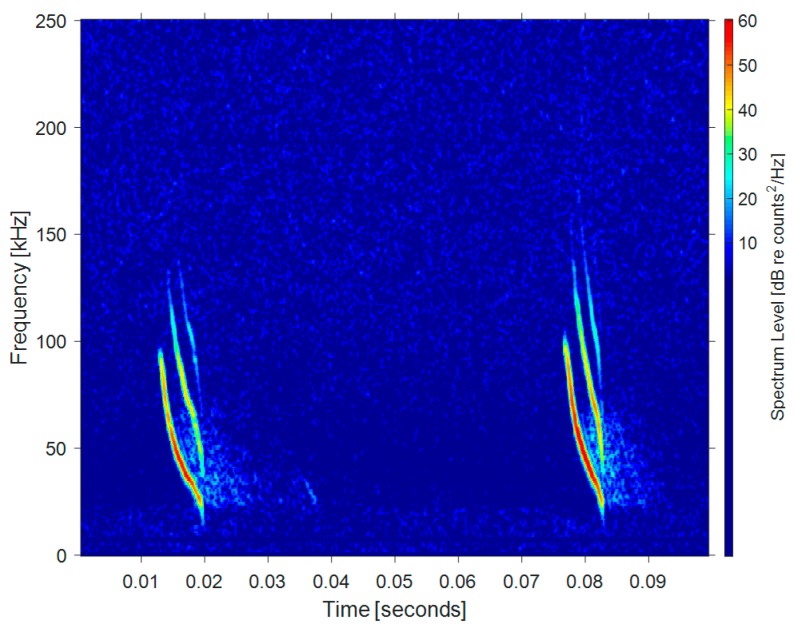
Harmonic components of a *Myotis nattereri* signal.

**Figure 5:**
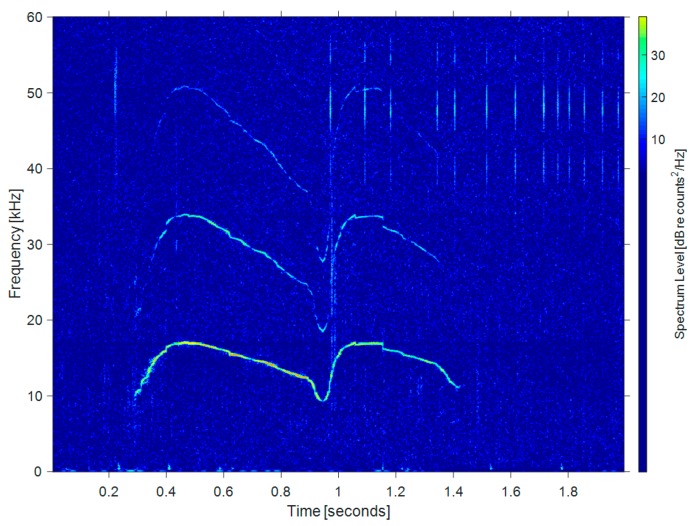
Bottlenose dolphin whistle and click signals.

Bats’ echo signals and dolphins’ whistle signals usually contain harmonics in one complete pulse, but this is not consistent across species or families [5,19]. The harmonics of some species are a multiple of the fundamental frequency [5,20,21,22]. Each harmonic has a specific frequency or range of frequencies. Bats increase their ability to discriminate objects by using directional sound waves with multiple harmonics. When bats prey on insects in complex environments, a series of harmonics may help them reduce clutter [5,19]. Figure 4 shows the time-frequency distribution of the harmonic components in the vocal signal of *Myotis nattereri*. Similar to bats, Figure 5 shows the whistles (left half) and clicks (right half) of a real bottlenose dolphin. It is generally believed that dolphins use whistle signals for inter-individual communication and high frequency short clicks to detect targets [5]. As can be seen, the whistle signals have obvious harmonic characteristics.

### 2.2. HFM-CW Multiharmonic Waveform Design

As we can see from the previous section, bat detection signals are mainly a combination of a frequency modulation signal and a constant frequency signal. However, the detection result of the CF and the FM signal will be different because of the Doppler effect generated by the moving target. The volume invariance of the ambiguity function is an important property that affects the signal waveform design. The ambiguity function of a narrowband signal is defined by Equation (1) [23], and for wideband by Equation (2) [24]. In order to achieve good time and velocity resolutions, which are the crucial factors for moving target detection, the ambiguity function of the signal should have a sharp main lobe and a low side lobe, which is often referred to as a pin-like function.

(1) $$\chi (\tau ,f_{d})=\int_{-\infty }^{+\infty }v(t)v^{*}(t+\tau )e^{-j2\pi f_{d}t}dt$$

where the *τ* is time delay associated with range difference, $f_{d}$ is the frequency shift, and *v(t)* is an energy normalized analytic signal.

(2) $$\chi_{w}(\tau ,s)=\sqrt{s}\int_{-\infty }^{+\infty }v(t)v^{*}[s(t+\tau )]dt$$

where *v(t)* is an energy normalized analytic signal, *s* is the compression factor associated with the velocity difference, and *τ* is the time delay associated with the range difference.

**Figure 6:**
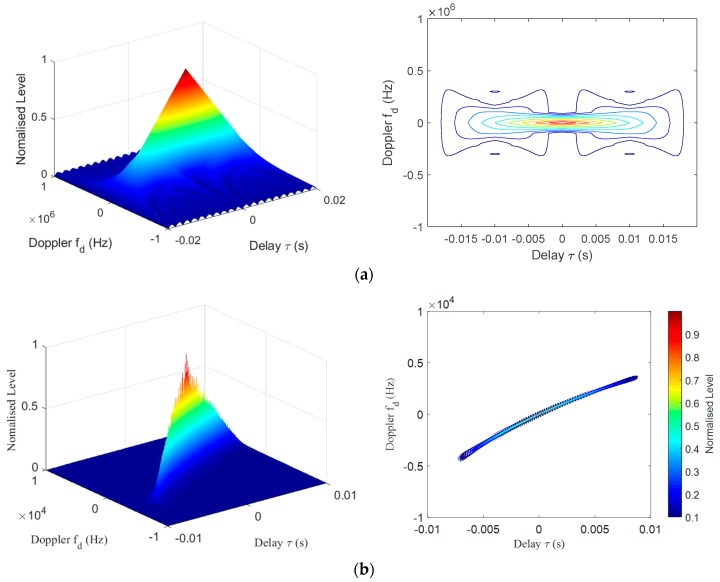
Continuous wave (CW) signal and hyperbolic frequency modulation (HFM) signal ambiguity: (**a**) Surface and contour plots of the CW signal ambiguity function. (**b**) Surface and contour plots of the CW signal ambiguity function.

Figure 6a shows the ambiguity function of the CW signal with frequency of 16 kHz and a pulse width of 0.02s. The function was calculated using Equation (1). It can be seen that the attenuation of the main lobe along the time axis is slow and the attenuation along the frequency axis is fast. That is to say, the long CW signal has a large time measurement blur, which is not conducive to the distance measurement of the target. When measuring the velocity of the moving target, the Doppler effect of the target echo occurs, and the frequency offset is generated. The CW signal can accurately reflect the offset, which is an advantage for accurate target velocity measurement. Therefore, the single frequency signal is also known as a Doppler-sensitive signal.

Figure 6b shows the ambiguity of the HFM signal, with a frequency range of 24–16 kHz for the down-chirp and a time width of 0.02 s. The function was calculated using Equation (2). It can be seen that it has a tilted, slowly decaying ridge in Figure 6b. The HFM signal has better time measurement performance than in the case of the CW signal. When the motion state of the target is known, the HFM signal can help determine the distance of the target by time measurement. When the target state is completely unknown, the timing of the signal is interfered by the target echo Doppler, and the accuracy cannot be guaranteed. That is to say, when measuring stationary targets, the ranging accuracy of the HFM signal should be higher than the CW signal.

**Figure 7:**
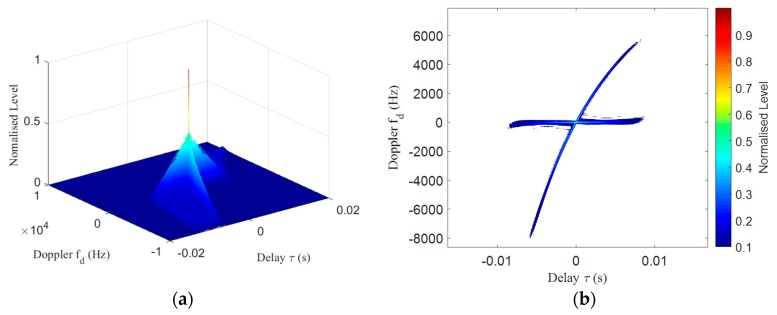
Contour plots of the HFM-CW signal ambiguity function: (**a**) Surface plot of the HFM-CW signal ambiguity function. (**b**) Contour plot of the HFM-CW signal ambiguity function.

According to the above analysis, combined with the characteristics of the bat sound signal, the HFM-CW detection signal was designed. The detection signal consists of a down-modulated HFM signal and a CW signal. Figure 7 shows the ambiguity function of the designed signal, with a 24–16 kHz HFM (0.01s) and a 16 kHz CW (0.01s) signal. The function was calculated using Equation (2). The main lobe of the combined detection signal is high, and the attenuation along the time and frequency axes is very fast, so it has the advantages of the CW and HFM signals and has excellent time and speed measurement performance. The CW component can be used to measure the Doppler shift of the target to determine the relative velocity. The HFM component can be used to measure the target range. The Doppler shift can also be used to compensate for the processing of the HFM signal for more accurate analysis of the echo.

**Figure 8:**
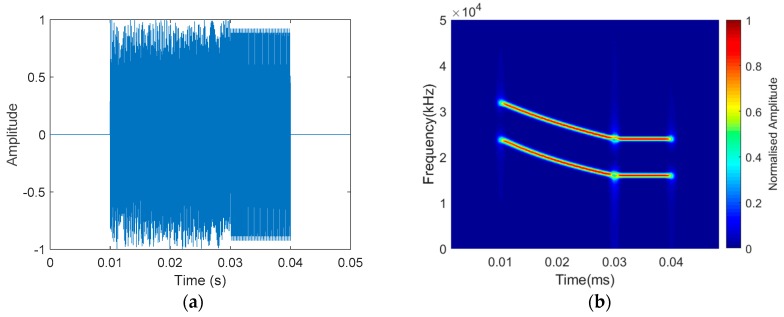
HFM-CW multiharmonic signal time-frequency analysis. (**a**) Time domain. (**b**) Time-frequency domain.

Considering the existence of harmonic information in the mammal sonar signal, we also added multiharmonic information to the detection signal and used multiscale matched filtering (detailed in Section 4) to obtain as much information as possible from the echo. The frequencies of the harmonic information were 32–24 kHz for the HFM information and 24 kHz for the CW information. We intended to use 8–16 kHz as the fundamental frequency, and 16–24 kHz and 24–32 kHz as harmonics, however, due to the band limitation of the transducer in the experiment, we had to use 16–24 kHz as the fundamental frequency. In order to facilitate subsequent processing, the harmonics in this paper were made to not overlap with the frequency of the fundamental frequency, and only harmonics twice the fundamental frequency were added. Figure 8 shows the short-time Fourier transform analysis of the HFM-CW multiharmonic signal.

## 3. Twin Inverted Multiscale Matched Filtering

### 3.1. Twin Inverted Matched Filtering

A twin inverted waveform was first proposed by Leighton, inspired by dolphins’ signals during when hunting prey [12,13,14,15]. Dolphins usually produce a pair of continues pulses, and it is believed that dolphins utilize these pulse pairs to discriminate between bubbles and fish when using bubble nets, in order to hunt and not be disturbed by bubbles. TWIPS is a process by which a source emits a time series signal [14,15]:

(3) p(t)=Γ(t)−Γ(t−Δ)

where the signal consists of a pulse Γ(t) followed by an identical pulse, except that it has an opposite polarity to the first pulse. If the environment is noise-free, the echoes from a linear scatterer will also consist of two parts, $\Gamma_{1}$ and $\Gamma_{2}$, which are evoked by Γ(t) and Γ(t−Δ) respectively, and we can find:

(4) $$p_{+}(t)=\Gamma_{1}+\Gamma_{2}=0$$

The explanation of Equation (4) is as follows [14]:

It is assumed that the pressure echo at the receiver can be regarded as the superposition of responses from individual scatterers. The received signal from an object that scatters linearly can be related to the emitted pulse *p(t)* via a convolution integral:

(5) $$p_{lin}(t)=\int_{-\infty }^{t}h_{in}(t-\tau )p(\tau )d(\tau )=H_{lin}[\Gamma (t)]-H_{lin}[\Gamma (t-\Delta )]$$

Here, the function (or impulse response) $h_{in}(t)$ incorporates the effects of propagation and the scattering from the object. The effects of propagation are assumed to be linear and time-invariant, however, in a realistic environment, the channel is usually time-variant, and this will affect the performance of the algorithm. The related problems here will be discussed later. The symbol $H_{lin}[]$ is used to represent the operator that computes. Since Γ(t) and −Γ(t−Δ) are identical, except for the inverted amplitude, $H_{lin}[\Gamma (t)]$ and $-H_{lin}[\Gamma (t-\Delta )]$, when considering a linear target, should also be identical, except the amplitudes are inverted, and therefore the superposition of this two part equation equals 0.

If there are nonlinear scatterers, $p_{+}(t)$ will have non-zero components. If the second part of the echo is subtracted from the first part of the echo:

(6) $$p_{—}(t)=\Gamma_{1}\;\;-\;\;\Gamma_{2}$$

Here, $p_{—}(t)$ indicates scattering from linear scatterers and scattering of the fundamental and odd-powered harmonics from nonlinear scatterers.

The characteristics above can be used to further discriminate of linear scatterers, like the seabed, and nonlinear scatterers, like bubbles. However, in this paper, we try to use this waveform to detect a moving target and suppress linear reverberation. Supposing there is a sound source with a constant position, the channel is noise-free, and time-invariant for a short time. If the sound source moved obviously, there would be a Doppler effect, which can cause extension and contraction in the frequency domain, and then the two pulses in the echo would not be matched accurately. So if the channel is noise-free and time-invariant, the p+ calculation will not be 0 from a linear target’s echo. Additionally, the transmission channel is ideal as long as the two pulses of TWIPS pass through the same channel, which means the constant time of all points in the transmission channel is longer than the pulse-width time, such that the two pulses in the echo will not mismatch due to the changes of channel. This means the channel does not change during the pulse-width time, or the change is not obvious. The sound source transmits a TWIPS signal, p(t), and gets an echo pair, namely, $\Gamma_{1}$ and $\Gamma_{2}$. Since the channel is time-invariant in a short period of time, the two echoes of TWIPS from a stable boundary will maintain an inverted relationship, therefore, $p_{+}(t)$ will be 0, just like the linear scatterers in Leighton’s paper [14]. However, the two echoes $\Gamma_{1}$ and $\Gamma_{2}$ from a moving target will be different, including differences in the echo structure and time delay of the two echoes, due to target movement. So, in this case, the $p_{+}(t)$ calculation would not equal 0. This is similar to the nonlinear target scenario in Leighton’s paper [14]. Consequently, this is only relative, where the “linear” and “nonlinear” in this paper are not like the real and nonlinear scatterers considered in Leighton’s paper [14]. However, the environmental scatters will be zero and therefore suppressed. The speed of target will definitely influence the performance of this method. Since the p+ calculation will highlight the movement of the target by using differences between the inverted pulses, if the target moves faster, the differences will become larger, and the output will be more evident.

**Figure 9:**
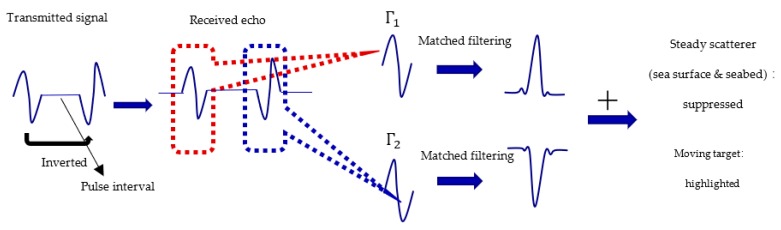
Twin inverted matched filtering process.

However, if the pulse is wide, the $p_{+}(t)$ result of TWIPS would also be long, which is not conducive to time measurement. As such, the matched filtering was first performed on two pulses before the p+ operation:

(7) $$P_{+}(t)=corr(\Gamma_{1},\Gamma (t))+corr(\Gamma_{2},\Gamma (t))$$

where *corr* indicates the correlation calculation. Under real conditions, the result of the p+ calculation cannot be 0, but because of the environmental noise and small changes in the channel, under the condition of an appropriate pulse interval, this method can effectively distinguish the moving target from steady scatters. The pulse interval determination will be introduced in Section 4.1. The main process of twin inverted matched filtering is presented in Figure 9.

### 3.2. Multiscale Matched Filtering

**Figure 10:**
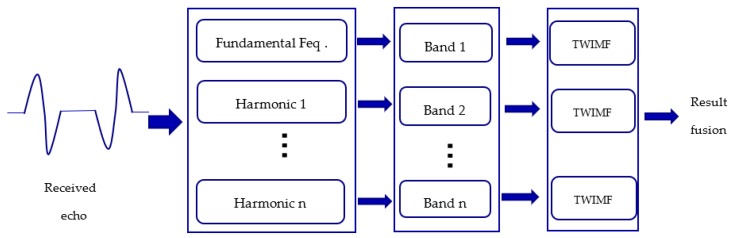
Twin inverted multiscale matched filtering process. TWIMF: Twin inverted matched filtering.

In Section 3.1, we introduced a Twin inverted matched filtering method to suppress reverberation and detect a moving target. The sound source transmits a time series signal, p(t), and here Γ(t) can be replaced by the signal designed in Section 2.2. However, how do we utilize this harmonic information? In this paper, a twin inverted multiscale matched filtering method is proposed as a solution. The idea is quite simple, where the echoes are divided into several bands according to the designed harmonic bands (in this paper, the bands are divided using simple FIR band-pass filters), and each band is processed separately using twin inverted matched filtering for the combination of results. The main process of this method is presented in Figure 10. By using this method, we can utilize as much information from the harmonic echoes as possible. Large bandwidth detection signals can bring more target structure information, and this is also why mammalian sonar uses broadband signals to detect targets. Besides, in an underwater environment, the channel usually fades frequency-selectively [25,26,27]. The frequency selective makes the underwater multipath channel more like a comb filter, where some frequencies will be filtered, and, therefore, this will lead to information loss. Additionally, in different frequency bands, the propagation loss is also different, usually in the higher frequency band, where the propagation loss is much higher, however, more target information can be obtained. Therefore, after the band division processing, the echo information of the target in different frequency bands can be obtained, and finally the detection results of every frequency band are combined. Here, in this paper, result combination is just the linear superposition of intensity. This method can effectively improve the detection performance.

## 4. Experiment Results and Discussion

### 4.1. Experiment Arrangement and Pre-Experiment Test

**Figure 11:**
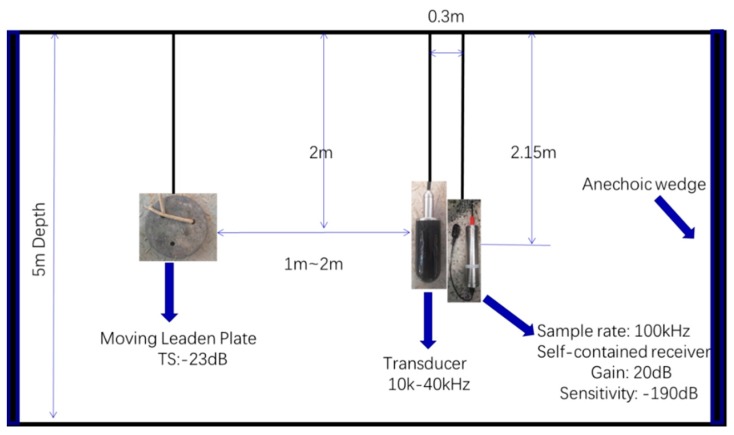
Experimental setup.

The experiment was conducted in the channel pool of Harbin Engineering University. The pool was about 30 m long, 8 m wide, 5 m deep, with anechoic wedges on both side of walls. The bottom of the pool was flat and made of concrete. As such, the reverberation was mainly from the water surface, bottom, weak flection of the anechoic walls, and higher order reflection from the bottom and surface. The transducer, receiver, and target were arranged according to Figure 11. The transducer bandwidth was 10–40 kHz, with two obvious resonance peaks at 18 and 27 kHz, and the transmitting response was higher at 10–20 kHz and lower at 20–40 kHz. The receiver was a self-contained hydrophone, with a −190 dB sensitivity and 20 dB amplification gain. The transducer and receiver were staggered to prevent reverberation between each other. The target was a leaden plate, with a diameter of 27 cm, and as such the largest target strength (i.e., facing the round part) was about −23 dB. Because the leaden plate was too heavy, it was difficult to keep its round face facing the transducer, so, during the movement, the leaden plate would rotate about a maximum of 30 degrees, so the target strength decreased. The target was at the same depth, with the transducer controlled to move back and forth at the distance of 1–2 m to the transducer to simulate a moving target, at a speed of about 20–40 cm/s.

Before using the twin inverted waveform, there was still a problem to be solved. How do we determine the pulse interval? The pulse interval should be long enough to prevent an overlap between the first echo and the second pulse, but it should not be too long, or else the channel will change too much. Here, the reverberation reduction time was first assessed by sending noise at the same band as the detection signal, calculating the average time when the reverberation energy declined by 60 dB (via the $t_{d}$ equation). The pulse interval should be longer than $t_{d}$, so that we can guarantee there is no overlap between the first pulse’s echo and the second pulse. The $t_{d}$ of the channel pool was assessed to be about 0.0238 s using this method.

**Figure 12:**
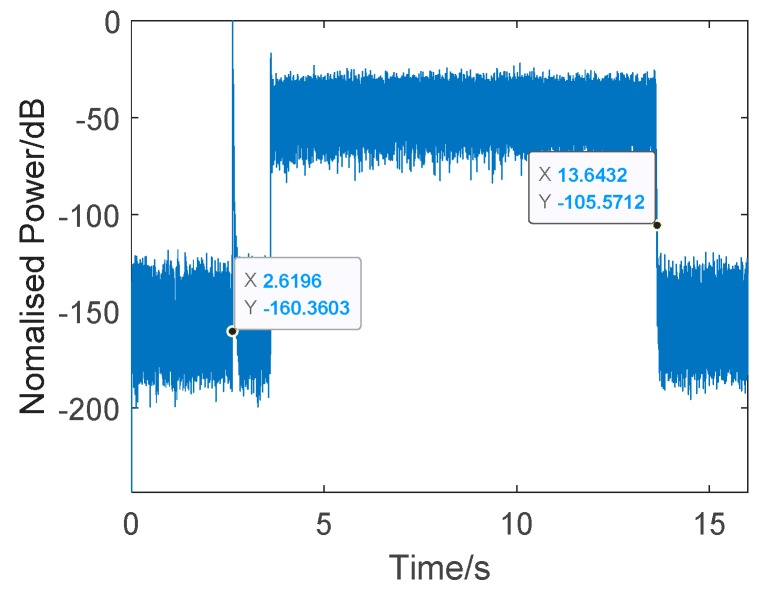
A single test result of reverberation reduction time.

Figure 12 shows a single test result. The transmitted signal, determined using Equation (8), consisted of a 16 kHz CW probe and band-pass Gaussian white noise ($noise_{fil}$, where the the pass band was 16–32 kHz). The CW probe was used as a synchronization head for accurate time measurement. The total length of the signal was 11 s, starting at 2.6196 s. The average energy of the received noise was about −44.5dB, and, at the time of 13.6432 s, the reverberation energy had decreased by 60 dB. Here, we calculated the reduction time to be about 0.0236 s. Considering the actual distance of the target set in the experiment, and the good channel conditions, the pulse interval was set as 0.1 s and pulse width was set to 1 ms to guarantee the target information was entirely acquired, even though the interval could be much shorter than 0.1 s.

**Figure 13:**
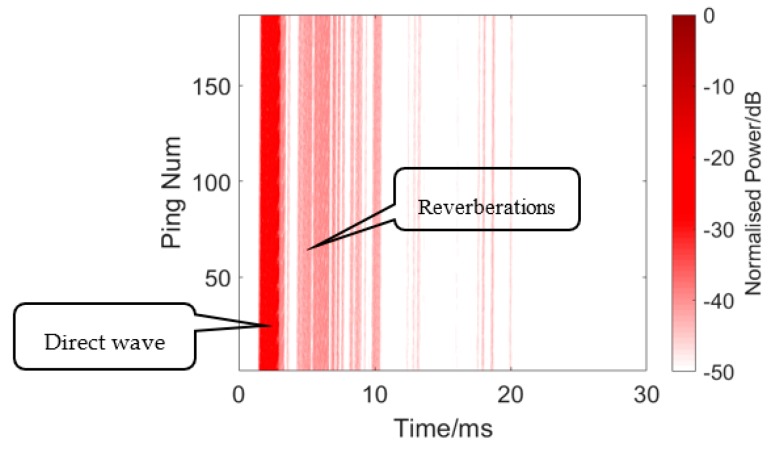
A rough channel estimation of channel pool.

**Figure 14:**
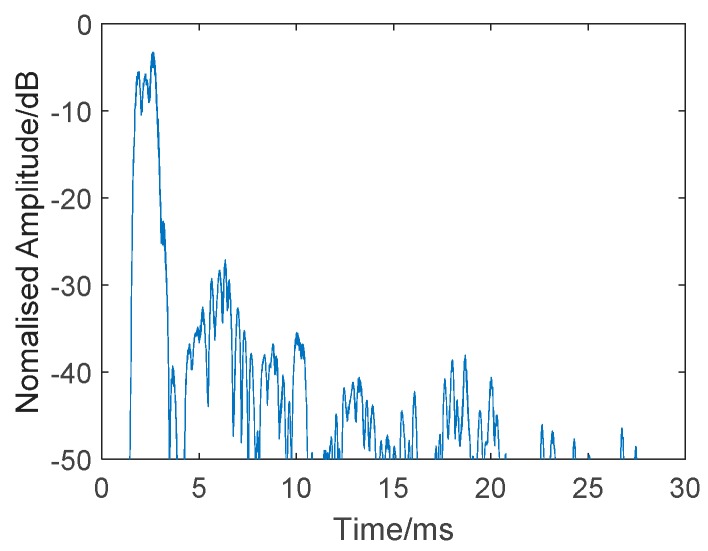
Single measurement of the channel.

Before the detection experiment, a simple channel estimation was also conducted by a correlation operation of the received echo and transmitted signal (24–16 kHz HFM signal), without a target. The result is presented in Figure 13. We can see strong reverberations after the direct wave. Figure 14 shows a single measurement of the channel.

(8) $$sig_{T60}=[\sin(2\pi \times 16e3\times t),zeros(1,0.99\times fs),noise_{fil}]$$

### 4.2. Detection Experiment Result

First, a controlled experiment was performed using traditional matched filtering. The detection signal used a 24–16 kHz HFM signal with a pulse width of 750 μs and signal interval of 0.1 s. The outputs of matched filter were further processed using Equation (9) to acquire a normalized power, where MF is the output of the matched filter.

**Figure 15:**
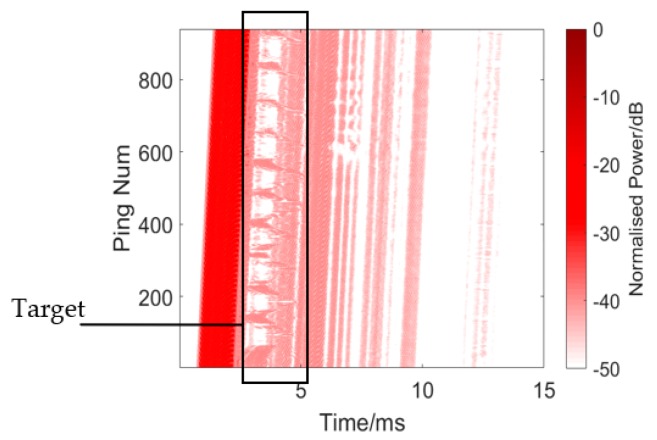
Echo processing using conventional matched filtering.

**Figure 16:**
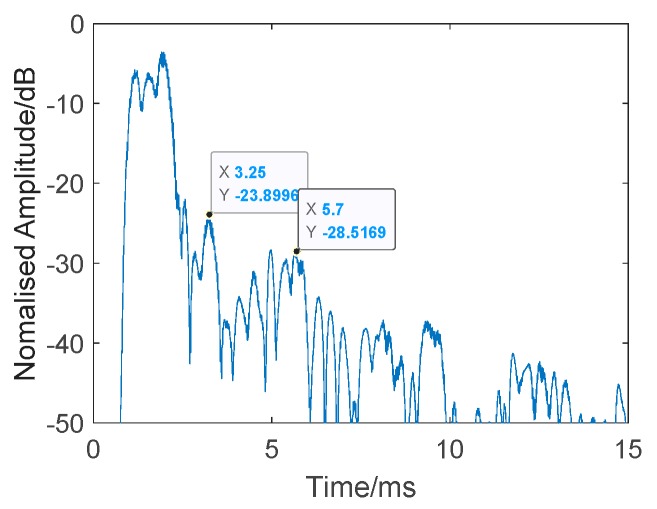
Single process of conventional matched filter. Target: −23.8996 dB. Reverberation: −28.5169 dB.

The results are presented in Figure 15 and Figure 16 and Table 1. Figure 16 shows the single process conventional matched filter. Table 1 shows 10 measurements of the target and reverberation amplitude. Since it is impossible to calculate an overall average due to the unstable state of the target, we did this instead by choosing 10 measurements and calculating the average strength difference between the target echo and reverberation. The weak target appears between the direct wave and the reverberations in the rectangle in Figure 15. However, when the target moves into the reverb wave, its echo is annihilated into the reverb. The average target echo intensity after processing was about 2.92 dB higher than the reverberant background intensity shown in Table 1.

(9) output=20∗log(|MF|/(max(|MF|))

**Figure 17:**
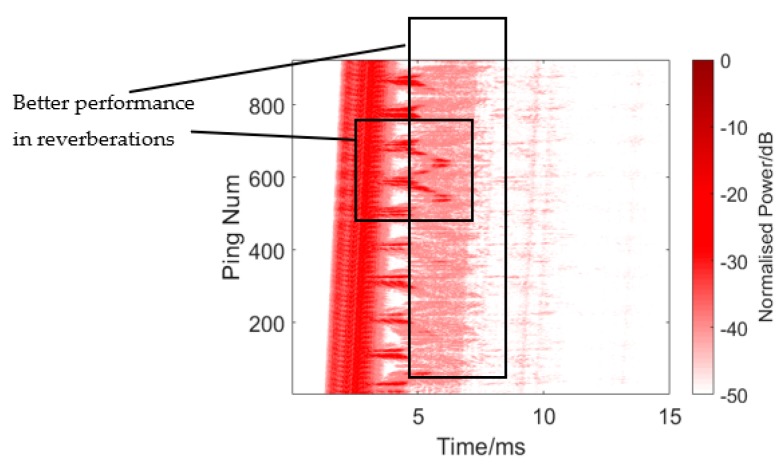
Echo processing using twin inverted matched filtering. In the right rectangle, the strong reverberations were suppressed. The vertical line is the multiple reflection of boundaries (pool bottom and surface mainly) and the vaguely curved line is the multiple reflection of the target.

**Figure 18:**
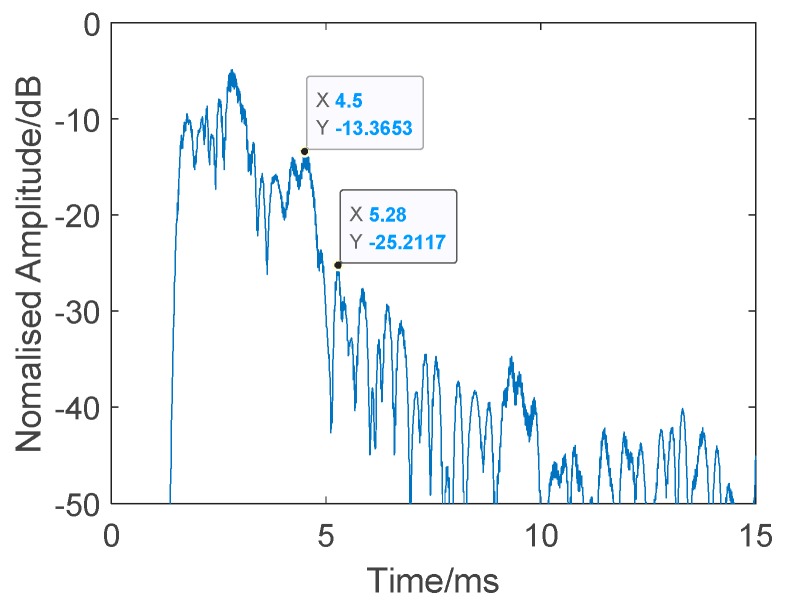
Single processing of twin inverted pulse sonar (TWIPS)-matched filtering without harmonics. Target: −13.3653 dB. Reverberation: −25.2117 dB.

The twin inverted matched filter was applied to process the echo. The detection signal used here was a HFM-CW (a 24k-16kHz HFM and a 16kHz CW) twin inverted signal without harmonics, where the pulse width was 1 ms, the interval of the twin pulse was 0.1 s, and the signal interval was also 0.1 s, which was convenient for subsequent processing. The result is presented in Figure 17 and Figure 18 and Table 2. Figure 18 shows a single process of TWIPS-matched filtering. Table 2 shows 10 measurements of the target and reverberation amplitude. Although there are still some messy reverberations, the strong reverberations were suppressed when compared with the results shown in Figure 15, and, at the same time, the target echo was highlighted, where the intensity was higher than the reverberation. However, there are still some problems, such as when the target moves into the messy reverb, where it will sometimes still be difficult to distinguish between reverbs and echoes. Sometimes the target can be detected in the reverb, for example, as in the left rectangle area in Figure 17. Overall, this method achieves better detection performance than the traditional matched filtering method, the average target echo intensity after processing was about 14.28 dB higher than the reverberant background intensity shown in Table 2.

Finally, the twin inverted multiscale matched filtering experiment was conducted. The detection signal waveform was the twin inverted HFM-CW multiharmonic waveform designed in Section 2.2, which consisted of the fundamental frequency (a 24–16 kHz HFM and a 16 kHz CW), and a double frequency harmonic (32–24 kHz HFM and 24 kHz CW). As mentioned before in Section 2.2, due to the band limitation of the transducer in the experiment, we had to use 16–24 kHz as the fundamental frequency instead of 8–16 kHz. The width of a single pulse was 1 ms (HFM 750 μs and CW 250 μs), the interval of the twin pulse was 0.1 s. and the signal interval was also 0.1 s. The sound source level was about 176 dB.

**Figure 19:**
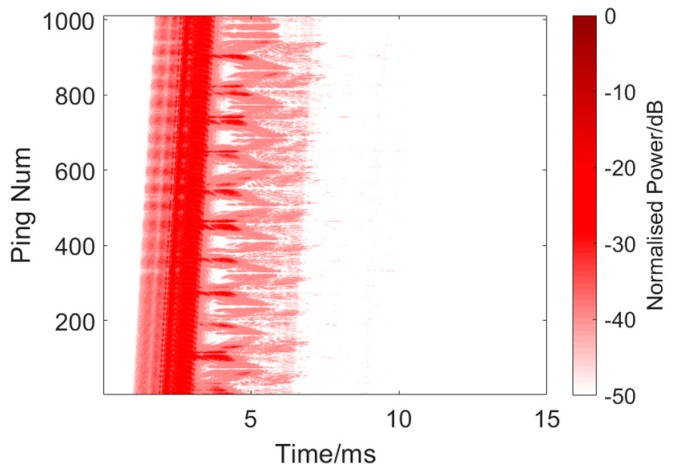
Twin inverted multiscale matched filtering experiment result.

**Figure 20:**
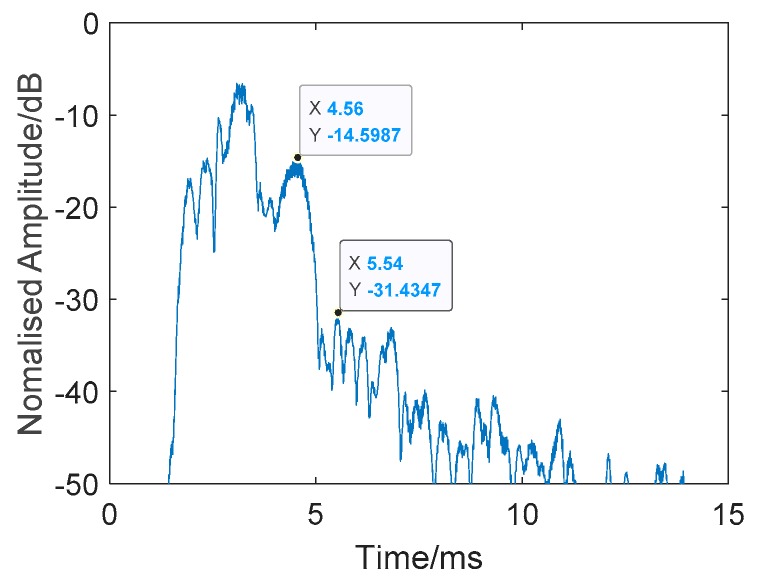
Single processing of twin inverted multiscale matched filtering. Target: −14.5987 dB. Reverberation: −31.4347 dB.

The results are presented in Figure 19 and Figure 20 and Table 3. Figure 19 shows a single process of signal processing using twin inverted multiscale matched filtering. Table 3 shows 10 measurements of target and reverberation amplitude. Through the combination of information found in the two frequency bands, the target echo was further strengthened, and the reverberation was further suppressed. As can be seen from the figure, the method has been able to clearly distinguish the target from the reverberations. The average target echo intensity after processing was about 19.86 dB higher than the reverberant background intensity shown in Table 3.

### 4.3. Discussions

By comparing the experimental results, we can conclude that the moving target detection method designed in this paper can achieve good performance. Compared with the traditional detection signal and matching filtering methods, the method of this paper can effectively suppress stable strong reverberation and highlight a moving target. Under the experimental conditions of this article, the target reverberation intensity increased by about 16.9 dB. However, there are still several issues in the experimental results that need to be discussed.

The first problem concerns the messy reverberations shown in Figure 17 and Figure 19. Are these present because the twin inverted method did not suppress the reverberation very well? Due to the experimental conditions, the target was close to the receiver and transducer, which results in second order (or even higher) reverberations between the transducer and the target, as well as between the receiver and the target. The higher order reverberations will interfere with the strong reverberations from the surface and bottom, causing changes in the waveform of the strong reverberations, eventually affecting the reverberation, suppressing the performance of the twin inverted matched filtering method.

**Figure 21:**
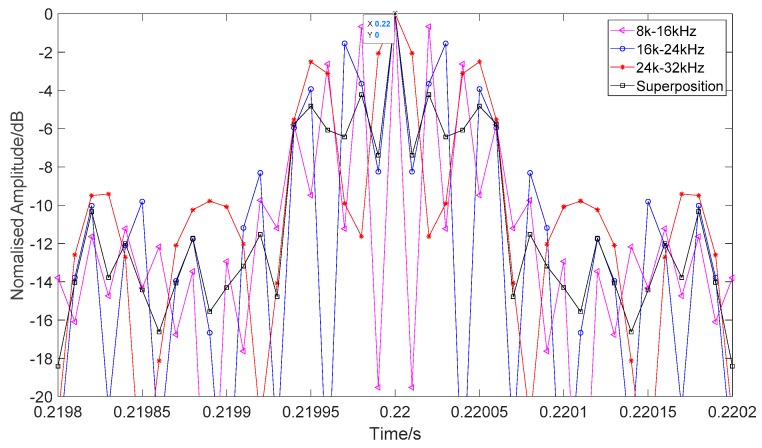
Matched filter outputs of different frequency bands.

Another problem is determining whether the matched filter outputs of the different frequency bands affect the target detection result. Since the multiharmonic signals in our paper were processed separately in different frequency bands, we simulated 16–8 kHz, 24–16 kHz, and 32–24 kHz down-chirp HFM-CW signals (HFM 0.015s and CW 0.005s, and 0.2 s left as blank on both sides) and used the matched filters separately. The results (interception near the peak) are shown in Figure 21. Although the signals are in different frequency bands, their peak points are all at 0.22 s. The frequency band does not affect the position of the matched filter peak. The problem is that the side lobes of the matched filter output of a single band are very high. The highest side lobes of 8–16 kHz, 16–24 kHz, and 24–32 kHz were only 0.67 dB, 1.55 dB and 2.5 dB lower than the main lobe, respectively. However, after superposition, the highest side lobe value was significantly reduced to −4.22 dB. This is mainly because of the smoothing effect of multiband superposition. According to the simulation results, we can conclude that superposition of different bands could weaken the side lobe effect and improve the detection performance, while, at the same time, the accuracy of time measurement is guaranteed, which is crucial to the subsequent TWIPS process, because the two echoes of TWIPS need to correspond to each other. We believe that with more subbands, the more obvious the smoothing effect will be. Although the matched filter output of different frequency bands is different, the main lobe information is mainly the same, and the differences in side lobes are smoothed by superposition. Therefore, we think that different matching filtering results of different frequency bands will not affect the detection results obviously, especially when there are many subbands. However, when there are few subbands, for example, only two, we think this will influence the detection performance. However, this is only an ideal analysis. In realistic application, the environment is much more complicated. In our experiments, we only used 2 bands, due to the transducer band limitation, so we are temporarily unable to verify our work with experimental data. We will try to verify the impact of more harmonics on this method in future work.

There are several issues that have not been addressed due to the limited experimental conditions, but these can be studied further in the future. The first issue concerns the linearity and nonlinearity of the target. In the experiment in this paper, the leaden plate was used as the target, which is a strongly linear target, with a strong echo. If the target is not strongly linear or if it is a nonlinear target, the results could be different. The second issue concerns the experimental environment. Further experiments should be conducted under real water conditions. In this paper, the experimental site was in an indoor pool, where there is no fluctuating water surface nor complex seabed sediment. Further experimentation is needed to determine whether the channel changes will have great influence on the method within the pulse interval time of the twin inverted pulse. The third issue concerns the impact of the random (uncontrolled) movements of the sound source on the performance of the proposed approach. In the experiment in this paper, the movement of the sound source was slight and can be ignored. Finally, only a HFM-CW signal was tested in this paper, and the performances of other signals need to be considered and tested in the future as well.

## 5. Conclusions

In this paper, a bioinspired twin inverted multiscale matched filtering method was proposed and used for detecting moving target in reverberations. The method mainly consists of the twin inverted HFM-CW multiharmonic detection signal and a twin inverted multiscale matched filtering echo processing algorithm. An indoor channel pool experiment was conducted to test the performance of the proposed method. After comparing the experimental results with the conventional matched filtering method, we can conclude that the moving target detection method designed in this paper can achieve good performance. The method of this paper can effectively suppress stable strong reverberations and highlight a moving target. Under the experimental conditions of this article, the target reverberation intensity of the proposed method is about 16.9 dB higher than with matched filtering, and 5.58 dB higher than with TWIPS-matched filtering without harmonics.

## Figures and Tables

**Table 1:** Ten measurements of target and reverberation amplitude from Figure 15.

Ping Number	Target Amplitude (dB)	Reverberation Amplitude (dB)
40	−25.3703	−27.5527
140	−23.8996	−28.5169
230	−25.9000	−28.7457
320	−23.8896	−28.1379
405	−21.1968	−27.7738
480	−25.8207	−28.7363
570	−24.1978	−28.3268
655	−27.9686	−28.4560
740	−28.2686	−28.6865
840	−27.3996	−28.1339
**Average**	−25.3911	−28.3067
**Average Difference**: 2.9156 dB		

**Table 2:** Ten measurements of target and reverberation amplitude from Figure 17.

Ping Number	Target Amplitude (dB)	Reverberation Amplitude (dB)
32	−10.0834	−24.2135
110	−8.0012	−26.7511
200	−13.3653	−25.2117
307	−13.7349	−23.3996
415	−6.9769	−25.3489
500	−18.4009	−23.6974
590	−8.1706	−27.5688
693	−15.0068	−20.496
772	−5.8254	−25.7930
862	−4.7159	−24.6019
**Average**	−10.4281	−24.7082
**Mean Difference**: 14.2802 dB		

**Table 3:** Ten measurements of target and reverberation amplitude from Figure 19.

Ping Number	Target Amplitude (dB)	Reverberation Amplitude (dB)
20	−19.1512	−38.5618
100	−10.5650	−34.9523
187	−15.8462	−34.0641
275	−14.8269	−35.9625
452	−12.7934	−36.6704
544	−13.1177	−35.1534
652	−18.4892	−30.5824
730	−12.8362	−34.0829
805	−14.5987	−31.4347
905	−13.4853	−32.8446
**Average:**	−14.5710	−34.4309
**Mean Difference**: 19.8599 dB		
